# Acute Liver Failure Caused by *Amanita phalloides* Poisoning

**DOI:** 10.1155/2012/487480

**Published:** 2012-07-03

**Authors:** Luca Santi, Caterina Maggioli, Marianna Mastroroberto, Manuel Tufoni, Lucia Napoli, Paolo Caraceni

**Affiliations:** ^1^U.O. Semeiotica Medica, Department of Clinical Medicine, Alma Mater Studiorum University of Bologna, Via Albertoni 15, 40138 Bologna, Italy; ^2^S.S.D. Liver Transplant, Department of Clinical Medicine, Alma Mater Studiorum University of Bologna, Via Albertoni 15, 40138 Bologna, Italy

## Abstract

Mushroom poisoning is a relatively rare cause of acute liver failure (ALF). The present paper analyzes the pathogenesis, clinical features, prognostic indicators, and therapeutic strategies of ALF secondary to ingestion of *Amanita phalloides*, which represents the most common and deadly cause of mushroom poisoning. Liver damage from *Amanita phalloides* is related to the amanitins, powerful toxins that inhibit RNA polymerase II resulting in a deficient protein synthesis and cell necrosis. After an asymptomatic lag phase, the clinical picture is characterized by gastrointestinal symptoms, followed by the liver and kidney involvement. Amatoxin poisoning may progress into ALF and eventually death if liver transplantation is not performed. The mortality rate after *Amanita phalloides* poisoning ranges from 10 to 20%. The management of amatoxin poisoning consists of preliminary medical care, supportive measures, detoxification therapies, and orthotopic liver transplantation. The clinical efficacy of any modality of treatment is difficult to demonstrate since randomized, controlled clinical trials have not been reported. The use of extracorporeal liver assist devices as well as auxiliary liver transplantation may represent additional therapeutic options.

## 1. Introduction

Acute liver failure (ALF) can be caused by the ingestion of mushrooms containing exceptionally powerful hepatotoxins [1]. Among mushroom intoxications, the amatoxin syndrome is of primary importance because it accounts for about 90% of fatalities [2]. It is characterized by an asymptomatic incubation period followed by the gastrointestinal and hepatotoxic phases, leading eventually to multiorgan failure and death. 

Although the exact incidence of mushroom poisoning is not precisely estimated due to a presumably relatively high number of underreporting cases, amatoxin poisoning is a worldwide problem. Approximately 50–100 fatal cases are reported every year in Western Europe, being less common in the United States; however, cases of amatoxin poisoning from Africa, Asia, Australia, and Central and South America have been also described [1, 2]. 

**Figure 1:**
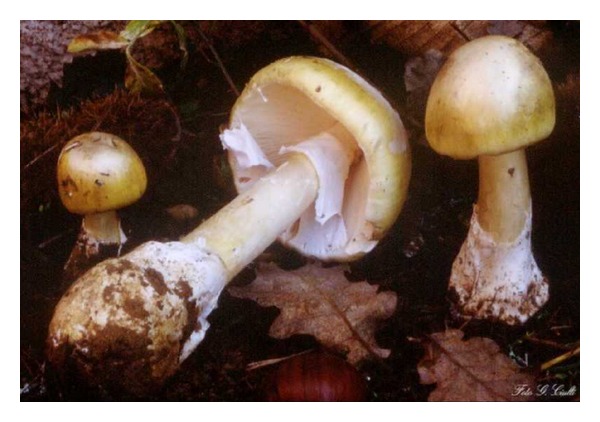
Image of the mushroom *Amanita phalloides*, commonly known as the death cap.

Amatoxin poisoning is caused by mushroom species belonging to three genera *Amanita*, *Galerina*, and *Lepiota*, with the majority of the fatalities attributable to *Amanita phalloides*, commonly known as the death cap [3] (Figure 1). 

Being the most common and deadly cause of mushroom poisoning, the present paper analyzes the pathogenesis, clinical features, prognostic indicators, and therapeutic strategies of ALF secondary to *Amanita phalloides* intoxication.

## 2. Mechanism of *Amanita* Toxicity and Pathogenesis of Liver Injury

The toxicity of *Amanita phalloides* is related to two distinct groups of toxins: phallotoxins and amatoxins. 

The phallotoxins consist of at least seven compounds, all of which have seven similar peptide rings. Their toxicity reside in the thiamide bond of the sulphur atom located on the indole ring. These toxins cause damage of the cellular membrane of the enterocytes and are therefore responsible of the initial gastrointestinal symptoms of nausea, vomiting, and diarrhea exhibited by almost all the patients. Even if phallotoxins are highly toxic to liver cells, they add little to the *Amanita phalloides* toxicity as they are not adsorbed from the intestine and do not reach the liver [4]. 

The amatoxins are bycyclic octapeptides, formed by at least nine different compounds [5]. Of the amatoxins, *α*-amanitin is the main component and along with *β*-amanitin is likely responsible for the toxic effect [6, 7]. They are not destroyed by cooking and can be still present in the mushroom after long periods of cold storage [8]. The lethal dose is very low: as little as 0.1 mg/kg body weight may be lethal in adults and this amount can be adsorbed even by ingesting a single mushroom. 

Amanitins are adsorbed through the intestinal epithelium and bind weakly to serum proteins. The liver is the principal organ affected, as it is the first organ encountered after absorption in the gastrointestinal tract [9]. Once in the liver, amanitins are transported by a nonspecific transport system into hepatocytes, producing an extensive centrolobular necrosis [4, 10]. About 60% of absorbed *α*-amanitin is excreted into the bile and is returned to the liver via the enterohepatic circulation [4, 11–15]. However, other organs, especially the kidney, are susceptible to their toxicity. Amatoxins are not significantly protein bound and are cleared from plasma within 48 h of ingestion [16, 17]. They are filtered by the glomerulus and reabsorbed by the renal tubules, resulting in acute tubular necrosis [18]. Finally, in animal and human post-mortem studies, cellular damage also has been found in the pancreas, adrenal glands, and testes [19, 20].

Amanitins directly interact with the enzyme RNA polymerase II in eucaryotic cells and inhibit the transcription, causing a progressive decrease in mRNA, deficient protein synthesis, and cell death. For this reason, metabolically active tissues dependent on high rates of protein synthesis, such as the cells of the gastrointestinal tract, hepatocytes, and the proximal convoluted tubules of kidney, are disproportionately affected. 

Among other potential toxic mechanisms, it has been proposed that alpha-amanitin acts in synergy with endogenous cytokines (e.g., tumor necrosis factor) and that this might cause cell damage through the induction of apoptosis [21]. 

## 3. Clinical Features and Diagnosis

The clinical picture due to *Amanita phalloides* poisoning can range from a mild subclinical presentation to a lethal fulminant course. As a result, not all patients with *Amanita phalloides* poisoning develop ALF and have a fatal outcome. The overall severity of the intoxication depends on the amount of toxin ingested and the time elapsed between ingestion and initiation of treatment. 

The clinical picture of *Amanita phalloides* intoxication is classically divided into four consecutive phases (Table 1).

(1) *Lag Phase*. As the toxins are not irritating by themselves, the initial phase is characterized by the absence of any signs or symptoms. The incubation time goes from 6 to 40 hours with an average of about 10 hours. It is important for an early diagnosis to suspect amatoxin intoxication in any case of a relatively prolonged latency period between mushroom ingestion and onset of symptoms, since other toxic mushrooms that do not cause liver involvement usually induce gastrointestinal symptoms much earlier, 1-2 h after ingestion [1, 2, 4, 10, 21].

(2) *Gastrointestinal Phase*. This phase is characterized by nausea, vomiting, crampy abdominal pain, and severe secretory diarrhea. Both diarrhea and emesis may become grossly bloody. This gastroenteritic phase may be severe enough to result in acid-base disturbances, electrolyte abnormalities, hypoglycemia, dehydration, and hypotension. This second stage lasts 12 to 24 h. After few hours, the patient seems to be clinically improving, if correction of the dehydration has been achieved. Liver and kidney function tests are usually normal at this point of the illness. If the association with toxic mushrooms is not made, these patients may be erroneously diagnosed with gastroenteritis and discharged home if hospitalized [1, 2, 4, 10, 21].

(3) *Apparent Convalescence*. 36–48 h after ingestion, signs of liver involvement may appear. In this third stage, despite the apparent improvement of gastrointestinal symptoms, the effects of toxins are damaging both the liver and kidneys, resulting in a progressive deterioration of liver enzyme tests with an increase of serum transaminases and lactic dehydrogenase. Clinical evidences of liver involvement ultimately develop with the onset of jaundice.

(4) *Acute Liver Failure*. In the last phase, the transaminases rise dramatically and liver and renal function deteriorate, resulting in hyperbilirubinemia, coagulopathy, hypoglycemia, acidosis, hepatic encephalopathy, and hepatorenal syndrome [22]. Multiorgan failure, disseminated intravascular coagulation, mesenteric thrombosis, convulsions, and death may result within 1–3 weeks after ingestion [23]. In contrast, in those patients with a favourable outcome, a rapid improvement in liver function tests occurs, followed by a full recovery and restoration of a normal quality of life.

Diagnosis is based on a careful assessment of history and clinical manifestations and can be confirmed by laboratory tests. The first task is to link the clinical presentation with mushroom ingestion, as the association may be obscured by the delay between symptom onset and the mushroom meal. When interviewing patients or the patient's relatives suspected of suffering from mushroom poisoning, physicians must obtain a detailed history concerning the ingestion. Key questions include the description of the eaten mushroom, the environment from which it was harvested, the number of different types of mushrooms ingested, the storage before consumption, the preparation before ingestion, the onset of similar symptoms in people who have eaten the same mushroom and the time frame between the mushroom ingestion and the onset of symptoms. Amanitins are resistant to heat and are still active after long periods of storage. Thus, in contrast to other toxins or bacterial contamination, cooking or prolonged cold storage may exclude other causes of mushroom intoxication, but not poisoning due to *Amanita phalloides* [10, 24]. 

Analysis of amatoxin levels in serum is not available for routine use in the clinical setting. The only specific laboratory test available is the detection of amatoxins in the urine. The role of this analysis is to confirm or exclude the diagnosis, not to grade the severity. We can use different methods of analysis (RIA, ELISA, HPLC), which are highly sensitive, without false negatives if performed in the first 48 h after ingestion [23, 25]. These procedures for alpha-amanitin urine are quite diffuse and not available only in specialized centers. Unfortunately, longer times can invalidate the accuracy of urine analysis. Furthermore, the relationship between the urinary concentration of *α*-amanitin and the severity of the liver damage is very weak [1].

Finally, the identification by a mycologist of any remaining mushrooms can be crucial for diagnosis.

## 4. Treatment Strategies

No specific amatoxin antidote is available. The clinical efficacy of any modality of treatment for amatoxin poisoning is difficult to demonstrate since randomized, controlled clinical trials have not been reported. 

The management of amatoxin poisoning consists of preliminary medical care, supportive measures, specific therapies, and liver transplantation. The specific treatments consist of detoxication procedures and chemotherapies. A complete analysis of the world experience in treatment of amatoxin poisoning was published in 2002 by Enjalbert et al. [2].

### 4.1. Preliminary Medical Care

Preliminary medical care consists of gastrointestinal decontamination procedures. The effectiveness of these treatments is closely related to an early execution. Because of the long asymptomatic latency, the clinical utility of these measures seems to be quite limited. Data to support or exclude the use of the emesis induced by ipecac syrup administration are insufficient, as well as for the use of whole bowel irrigation. Gastric lavage should be considered only when it could be performed early after ingestion [2].

### 4.2. Supportive Measures

The first goal should be directed to treat dehydration, electrolyte abnormalities, and metabolic acidosis caused by the gastrointestinal phase of the intoxication. 

### 4.3. Specific Measures

#### 4.3.1. Detoxification Procedures

Detoxification procedures consist of two different approaches: the reduction of intestinal absorption and enhancement of excretion. 

(1) *Oral Detoxification*. Repeated activated charcoal administration should avoid reabsorption of the toxins due to their enterohepatic circulation, although there is no evidence that its use improves clinical outcome. Gastroduodenal aspiration through a nasogastric tube has been recommended as a sole technique or combined with activated charcoal to remove bile fluids and interrupt enterohepatic circulation, but the actual benefit of these procedures is not documented. If diarrhea has ceased, the use of cathartics is recommended [2, 21].

(2) *Urinary Detoxification*. Intense forced neutral diuresis is no longer recommended, with urinary output of 100–200 mL/h for 4-5 days being sufficient to increase the renal elimination of amatoxins.

(3) *Extracorporal Purification Procedures*. Treatment with the Molecular Adsorbent Recirculating System (MARS) has been recently described [26]. Although the real efficacy of this method, or that of the other liver support systems, should be analyzed in appropriate trials, their use may represent a potential additional option to treat patients with severe amanitina poisoning. MARS is a modified dialytic method that mimics the biological features of the hepatocyte membrane by transferring protein-bound and water-soluble toxic metabolites from the blood stream into a dialysate compartment via a special membrane. The method was shown to be efficient in improving liver function by continuously removing protein-bound substances [27]. However, it is generally accepted that extracorporeal decontamination treatment is useful only if started very early, soon after the gastrointestinal symptoms occur [28].

#### 4.3.2. Chemotherapies

According to retrospective data, most authors indicate that silibinin and N-acetylcysteine (NAC) may be effective in the management of patients with *Amanita phalloides* poisoning [1, 2, 4, 21]. Many other drugs were used in the past for amatoxin poisoning: antibiotics, antioxidants, thioctic acids, hormones, and steroids: all have been abandoned.

Silibinin, a water soluble silymarin derivate, competes with amatoxins for transmembrane transport and inhibits the penetration of amanitin into hepatocytes, thus having direct hepatoprotective effect [29]. Moreover, silibinin appears to affect also the secondary uptake in the liver mediated through an enterohepatic recirculation. 

Administration of silibinin is recommended if the patient is seen within 48 hours of ingestion. The doses are 20–50 mg/kg/day intravenously and treatment should be continued for 48–96 hours. Silymarin capsules may also be given in dose from 1.4 to 4.2 g/d orally [30, 31].

Penicillin G seems to have a similar mechanism of action, displacing amanitin from the binding to plasma protein and thus promoting its excretion and preventing its hepatic uptake [29]. Penicillin G is used in continuous intravenous administration of high doses of Na/K penicillin G (1,000,000 IU/kg for the first day, then 500,000 IU/kg for the next two days) [30]. Although combined treatment with silibinin and penicillin has been suggested, there are no clinical data to support that this approach is superior to monotherapy with silibinin [1]. 

Data suggesting hepatoprotection by antioxidants support the use of free radical scavengers, such as N-acetylcisteine (NAC), in the management of amatoxin intoxication [32]. NAC is used in many centers in patients with ALF not induced by paracetamol and its administration has been proposed also in cases of amatoxin poisoning although the data are quite limited. N-acetylcysteine is usually administered intravenously in 5% dextrose, but 0.9% saline may be also used. The suggested dosage is 150 mg/kg over 15 min intravenously, followed by 50 mg/kg over 4 hours intravenously, followed by 100 mg/kg over 16 hours intravenously. Infusion of the initial dose over 30 to 60 minutes (rather than 15 minutes) may reduce incidence of anaphylactoid reactions [33, 34].

### 4.4. Liver Transplantation and Prognostic Indicators

Amatoxin poisoning may progress into ALF and eventually death, if liver transplantation (LT) is not performed. On the basis of the available data, the mortality rate after *Amanita phalloides* poisoning ranges from 10 to 20% [2, 29, 30]. Patients with severe liver injury should be admitted to Intensive Care Unit connected to a liver transplant centre.

Two surgical options, orthotopic liver transplantation (OLT) and auxiliary partial liver transplantation (APOLT), have been developed. OLT is a well-established procedure requiring long immunosuppression to prevent graft rejection. Because some patients with partial hepatectomy and temporary support may have complete morphological and functional recovery of their own liver, APOLT can represent an alternative approach. In APOLT, only a portion of the native liver is removed and the remainder is left in situ; the transplant provides temporary assistance until the native liver recovers and the immunosuppression can be withdrawn. 

The major dilemma in patients with ALF is to find the right timing for transplantation. If the surgical procedure is performed too early, the patient could have survived without impaired quality of life. If the search for a liver graft starts too late, the patient may die before a suitable donor organ becomes available. Several sets of criteria to decide the timing of liver transplantation in patients with ALF have been proposed, although they are not universally accepted (Table 2). Since the number of patients with amatoxin poisoning evaluated for LT is quite small, the prognostic indicators are not clearly defined in this specific condition. 

The most widely used criteria for urgent LT in patients with ALF are those of the King's College Hospital described by O'Grady et al. [35] which include different parameters for paracetamol and nonparacetamol induced ALF. These criteria are based on prothrombin time (PT), age, etiology, time elapsing between appearance of jaundice and onset of encephalopathy, and bilirubin concentration. In contrast, the Clichy criteria for urgent LT are based on Factor V, age, and encephalopathy [36]. 

However, some of these criteria cannot be easily transferred in patients with amatoxin poisoning. Ganzert et al. [37] retrospectively analyzed the outcome of a large series of amatoxin intoxication cases and found that predictors of death were the prothrombin index in combination with the serum creatinine level on 3–10 days after ingestion. However, although the presence of hepatic encephalopathy is an absolute requirement for the diagnosis of ALF in King's and Clichy criteria, this clinical manifestation was not adequately investigated in the paper by Ganzert et al. because of “imprecise data in the patient's records” [37]. Thus, these authors proposed that a patient with amatoxin poisoning should be listed for urgent LT regardless of the presence of hepatic encephalopathy, if the prothrombin index is less than 25% and serum creatinine greater than 106 *μ*mol/L at the third day after ingestion. 

Also Escudié et al. [38], in a retrospective study including 27 patients admitted for *Amanita phalloides* poisoning, suggested that encephalopathy should not be an absolute prerequisite for deciding liver transplantation. Nonetheless, independently of any other variables, a decrease in prothrombin index below 10% of normal (INR > 6) 4 days or more after ingestion should lead to consider urgent LT. Interestingly, these authors proposed that an interval between the ingestion of toxic mushrooms and the onset of diarrhea shorter than 8 h should prompt an especially careful monitoring because of the high risk of fatal outcome.

Furthermore, data from Enjalbert et al. [2] in patients transplanted for amatoxin poisoning indicate that Factor V was below 20% in all cases except one.

Finally, it should be taken into account that most of studies on the efficacy of prognostic criteria for urgent LT in patients with ALF have been carried out in countries where graft is usually available within a short time. However, the waiting time on the emergency transplant list, if it exists, may be very prolonged in other parts of the world [10], and liver transplant may never be performed in others [39]. In these situations, the use of new therapies (i.e., MARS) could be useful as well as the availability of other surgical techniques, such as APOLT. 

## Figures and Tables

**Table 1:** Criteria for urgent liver transplantation in patients with ALF. Only Ganzert's criteria are developed specifically for Amanita phalloide poisoning.

Clichy's criteria	(a) Combination of a decrease in factor V below 30% of normal in patients over 30 years or below 20% of normal in patients below 30 years
	(b) Grade 3-4 en cephalopathy

King's College criteria for nonparacetamol causes	(a) Prothrombin time over 100 s (*≈*INR over 7) or
	(b) At least three of the following criteria:
	(i) prothrombin time over 50 sec (INR over 3.5),
	(ii) serum bilirubin over 300 *μ*mol/L,
	(iii) age below 10 years or over 40 years,
	(iv) an interval between jaundice and encephalopathy over 7 days,
	(v) drug toxicity

King's College criteria for paracetamol causes	(a) Arterial pH below 7.3 or arterial lactate above 3 mmol/L after adequate fluid resuscitation
	*or*
	(b) Concurrently, serum creatinine above 300 *μ*mol/L, INR above 6.5 and encephalopathy of grade 3 or more

Ganzert's criteria	(a) A decrease in prothrombin index below or equal to 25% of normal at any time between day 3 and day 10 after ingestion
	*in association with*
	(b) Serum creatinine over or equal to 106 *μ*mol/L within the same time period

Escudie's criteria	Prothrombin index below 10% of normal (INR of *≈*6) 4 days or more after ingestion

**Table 2:** Clinical phases of the Amatoxin syndrome.

Phases	Onset from ingestion	Symptoms and signs
Stage 1. Lag phase	0–24 h	Asymptomatic
Stage 2. Gastrointestinal phase	6–24 h	Nausea, vomiting, crampy abdominal pain, and severe secretory diarrhea
Stage 3. Apparent convalescence	24–72 h	Asymptomatic, worsening of hepatic and renal function indices
Stage 4. Acute liver failure	4–9 days	Hepatic and renal failure → multiorgan failure → death
